# Supplementary material for: Lacrimal Gland Epithelial Cells Shape Immune Responses through the Modulation of Inflammasomes and Lipid Metabolism
Source: Int J Mol Sci. 2023 Feb 21;24(5):4309. doi: 10.3390/ijms24054309 (PMC10001612; doi:10.3390/ijms24054309)
Supplement: Supplementary file 1 [file ijms-24-04309-s001.zip › ijms-2156699-supplementary/ijms-2156699 figure/ijms-2156699-figure.pdf]

Description of supplemental tables

Table S1: Expression data for the genes belonging to the Gene Ontology process entitled "Interleukin-1 production (GO:0032612) in regenerating lacrimal glands. Timepoints were compared to the uninjured control. Expression fold-changes (FC) that meet the threshold $log_2(FC) = \pm log_2(1.5)$ are formatted in red. Significant adjusted p values (p-adj < 0.05) are formatted in orange/brown. Genes that meet all thresholds ($log_2(FC) = \pm log_2(1.5)$, p-adj < 0.05) on both days 1 and 2 are highlighted in yellow.

Table S2: Expression data for the genes that belong to the Gene Ontology process entitled "Interleukin-1 production (GO:0032612) in lacrimal glands of *BALBc* (control) and *NOD.H2*^{b} (diseased) male mice. Mice were processed at 2, 4 or 6 months old and diseased glands were compared to (sheet 1) healthy controls (sheet 2) other age groups, as indicated. Expression fold-changes (FC) that meet the threshold $log_2(FC) = \pm log_2(1.5)$ are formatted in red. Significant adjusted p values (p-adj < 0.05) are formatted in orange/brown. Genes that meet all thresholds ($log_2(FC) = \pm log_2(1.5)$, p-adj < 0.05) in the column comparing all *NOD.H2*^{b} and *BALBc* mice are highlighted in yellow.

Table S3: Expression data and description of the selected 66 genes upregulated on day 3 after lacrimal gland injury, compared to the uninjured control.

Table S4: Expression data and description of genes belonging to biological pathways related to lipid metabolism and altered in *NOD.H2*^{b} LGs compared to (sheet 1) healthy controls (sheet 2), other age groups as indicated. Data for the genes related to lipid metabolism and significantly upregulated on day 3 after acute injury was included in the table.

Supplemental Figures S1-S8

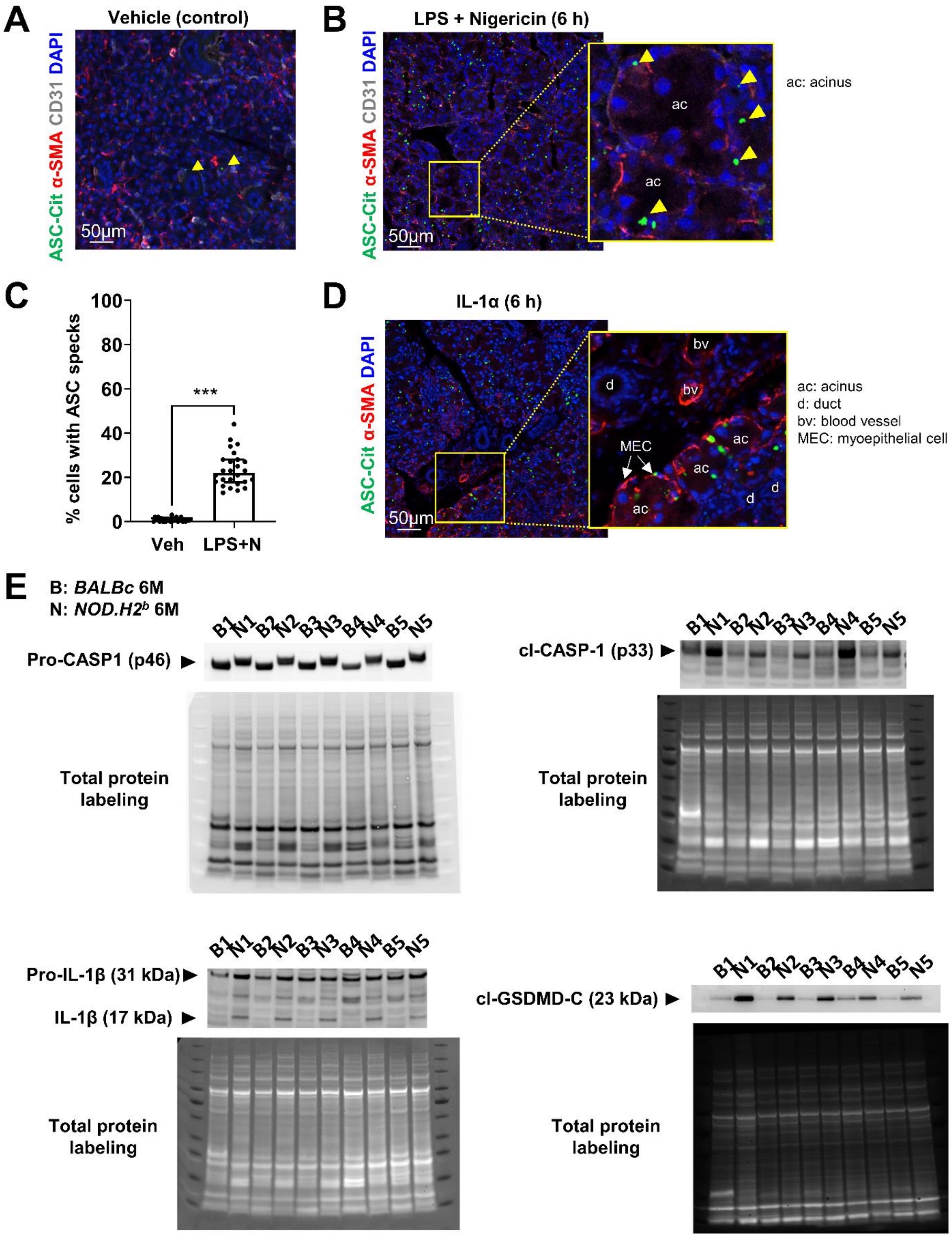

Fig. S1: (A-C) *R26^ASC-citrine^* mouse LGs (n=6 per group) were injected with (A) vehicle control, (B) LPS+nigericin and harvested 6 h after injury. (A, B) LG sections were analyzed for ASC specks formation (green) and immunostained for α-SMA (red) and CD31 (grey). Yellow arrowheads indicate ASC specks, which were found in great numbers in epithelial structures such as acini (see enlarged micrograph). (C) Percentage of cells displaying ASC specks after LPS+nigericin injection (median (IQR), Mann-Whitney test). Significant differences are represented as * if p value $p < 0.05$, ** if $p < 0.01$ and *** if $p < 0.001$. (D) LG sections from *R26^ASC-citrine^* mice injected with IL-1α and analyzed for ASC specks formation (green) 6 h after injury. The enlarged micrograph shows the presence of fluorescent specks in α-SMA+ MECs and acinar (ac) cells, while α-SMA+ blood vessels (bv) and α-SMA- ducts (d) do not have specks at this time-point. (E) Full western blot results for the analysis of CASP1, IL-1β and GSDMD proteins in the LGs of 6M *BALBc* and *NOD.H2^b^* mice (n=5 animals). Below each protein of interest is shown the total protein staining of respective membrane that was analyzed with ImageLab software to determine normalization factors.

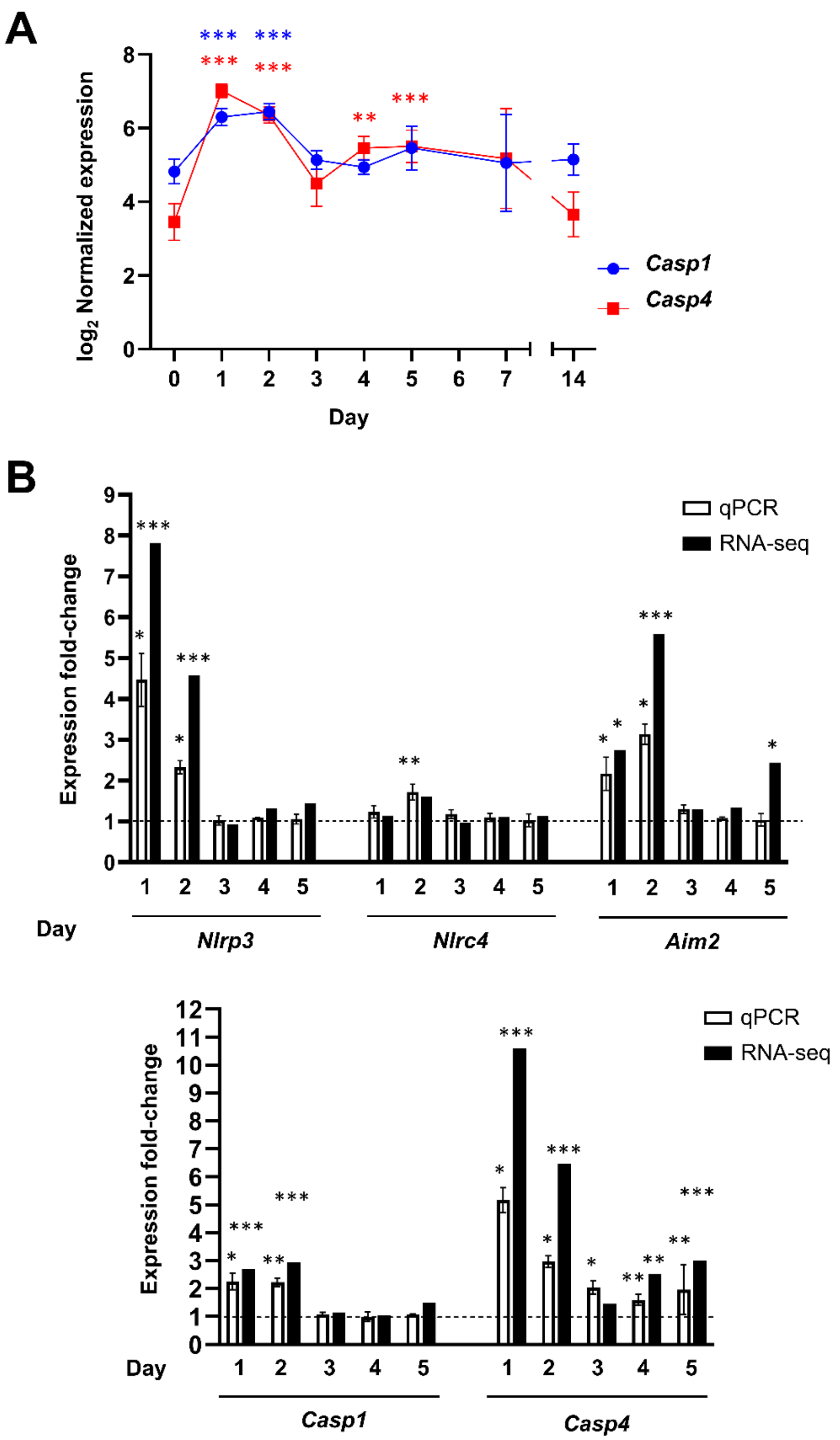

Fig. S2: Gene expression fold-changes for genes encoding (A) the inflammasome effectors CASP1 and CASP4 obtained by RNA-seq, (B) the inflammasome sensors NLRP3, NLRC4 and AIM2, as determined by RNA-seq (in black) or RT-qPCR (in white) analysis of LGs harvested at different time points after acute IL-1α injury. Error bars show mean (SD). Statistical significance of Fold Change compared to uninjured control (Day 0) is shown with: * P-value adj. < 0.05; ** P-value adj. < 0.01; *** P-value adj. < 0.001.

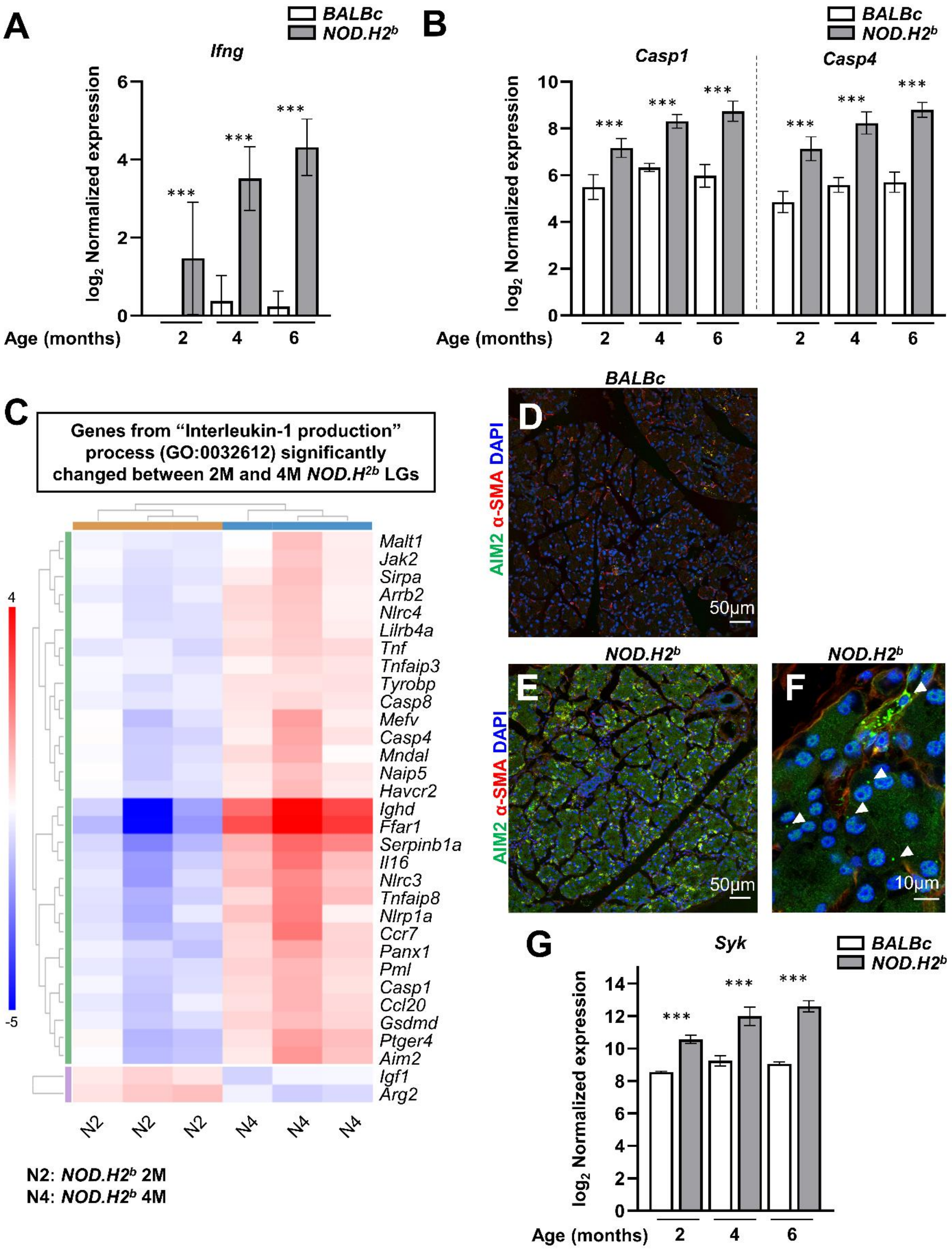

Fig. S3: (A-B, G) Normalized expression of genes from the pathway "interleukin-1 production" and significantly upregulated in LGs of *NOD.H2b* compared to *BALBc* mice. Error bars show the mean (SD). Statistical significance of $\log_2$ Fold Change compared to respective *BALBc* control is shown with: * P-value adj. < 0.05; ** P-value adj. < 0.01; *** P-value adj. < 0.001. (C) Expression heatmap of the genes from the Gene Ontology process entitled "Interleukin-1 production (GO:0032612) that are significantly changed between 2M and 4M *NOD.H2b* LGs (biological replicates are annotated N2 and N4, respectively). (D-F) Confocal images of LG cryosections from 3M (D) *BALBc* or (E-F) *NOD.H2b* mice stained for AIM2 (green) and α-SMA (red). Nuclei were counterstained with DAPI (blue).

Components of the protein-protein interaction network from the list of 219 genes upregulated on day 3 after acute injury

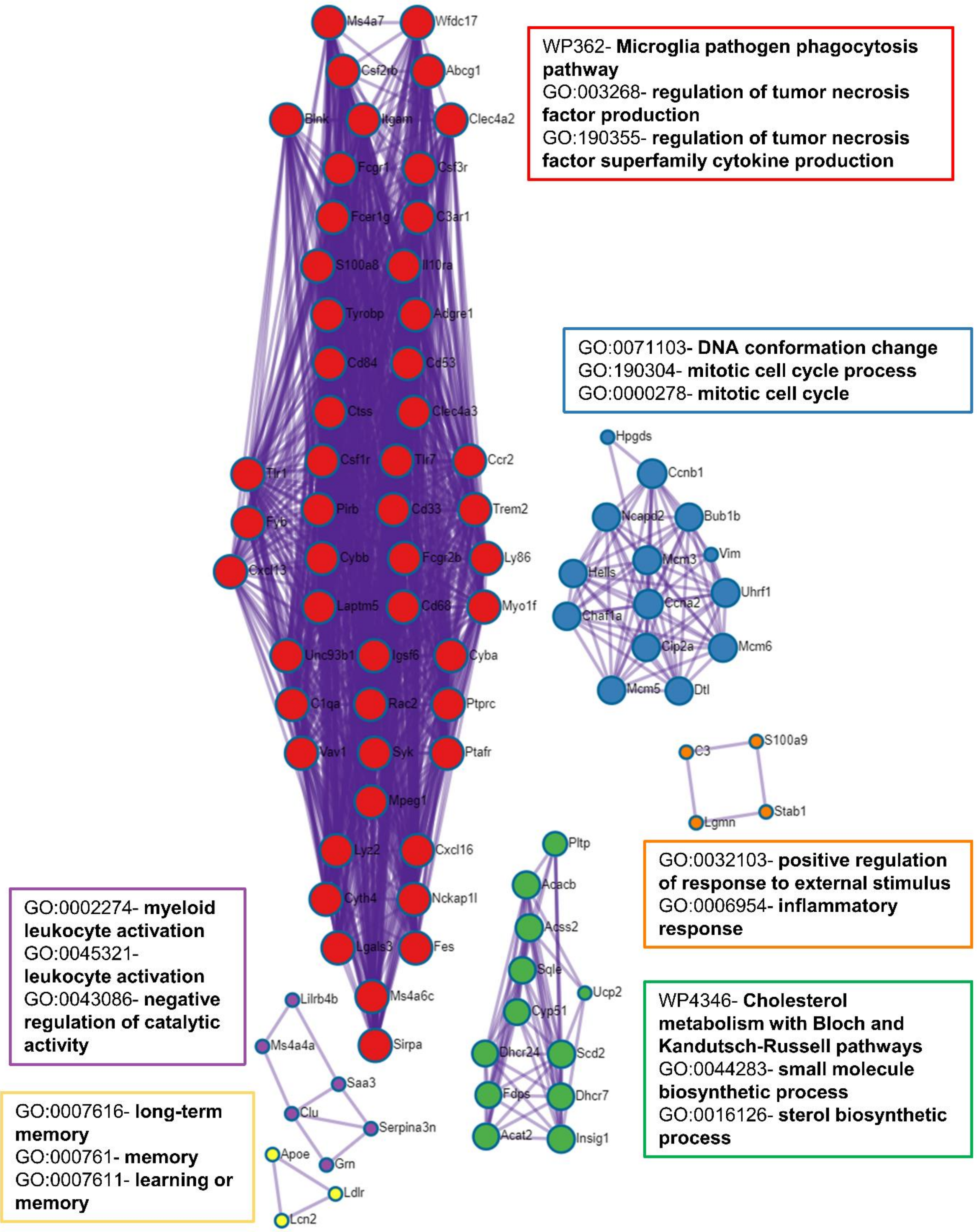

Fig. S4: Using Metascape, the network resulting from protein-protein interaction enrichment analysis of the 219 genes upregulated by RNA-seq on day 3 after LG acute injury was processed with Molecular Complex Detection (MCODE) algorithm10 to identify densely connected network components. Analysis evidenced 5 major networks colored by their identities. Gene Ontology (GO) enrichment analysis was applied to each MCODE network and the top three best p-value terms were retained for the description of the networks in respective colored frames.

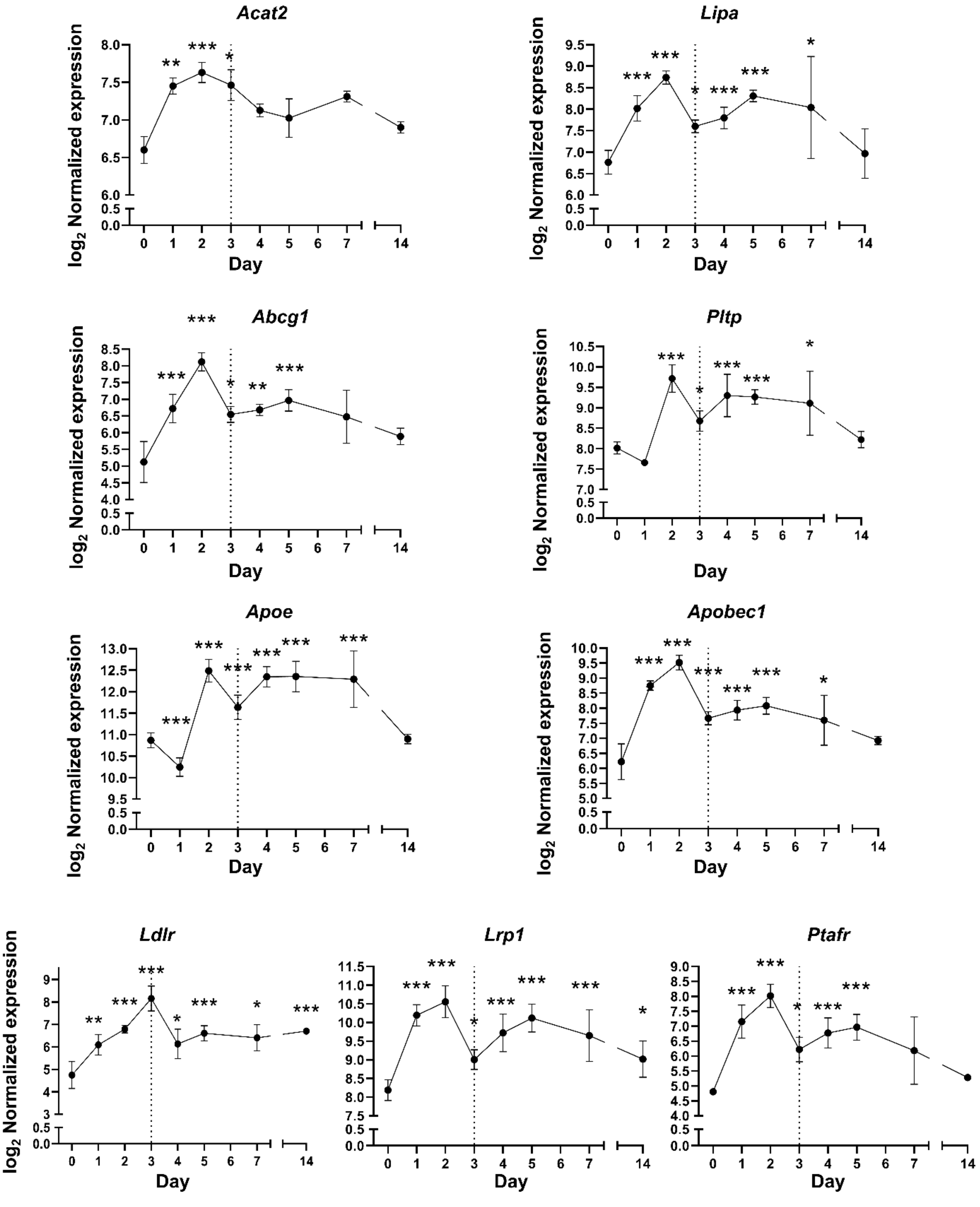

Fig. S5: Normalized mRNA expression of genes from the lipid metabolism pathways significantly upregulated on day 3 but reaching their maximum on day 2 after LG acute injury. Error bars show the mean (SD). Statistical significance of log_2 Fold Change compared to uninjured control (Day 0) is shown with: * P-value adj. < 0.05; ** P-value adj. < 0.01; *** P-value adj. < 0.001.

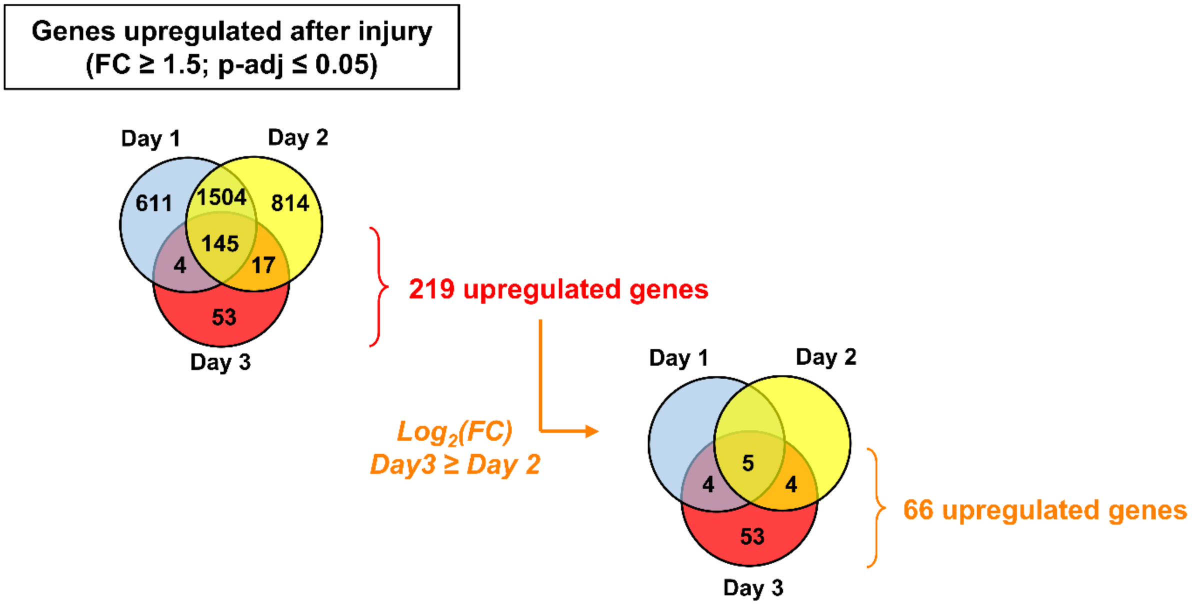

Fig. S6: The overlap between the genes upregulated (FC ≥ 1.5; p-adj < 0.05) on days 1, 2 and 3 after LG acute injury is shown by a Venn diagram. From the list of 219 upregulated on day 3, we retained all 53 genes specific to day 3 and those maintained/increased between day 2 and day 3 *i.e.* with $Log_2(FC_{Day3}) \geq Log_2(FC_{Day2})$ (relative to uninjured control). The distribution of the resulting 66 genes is shown on the second Venn diagram.

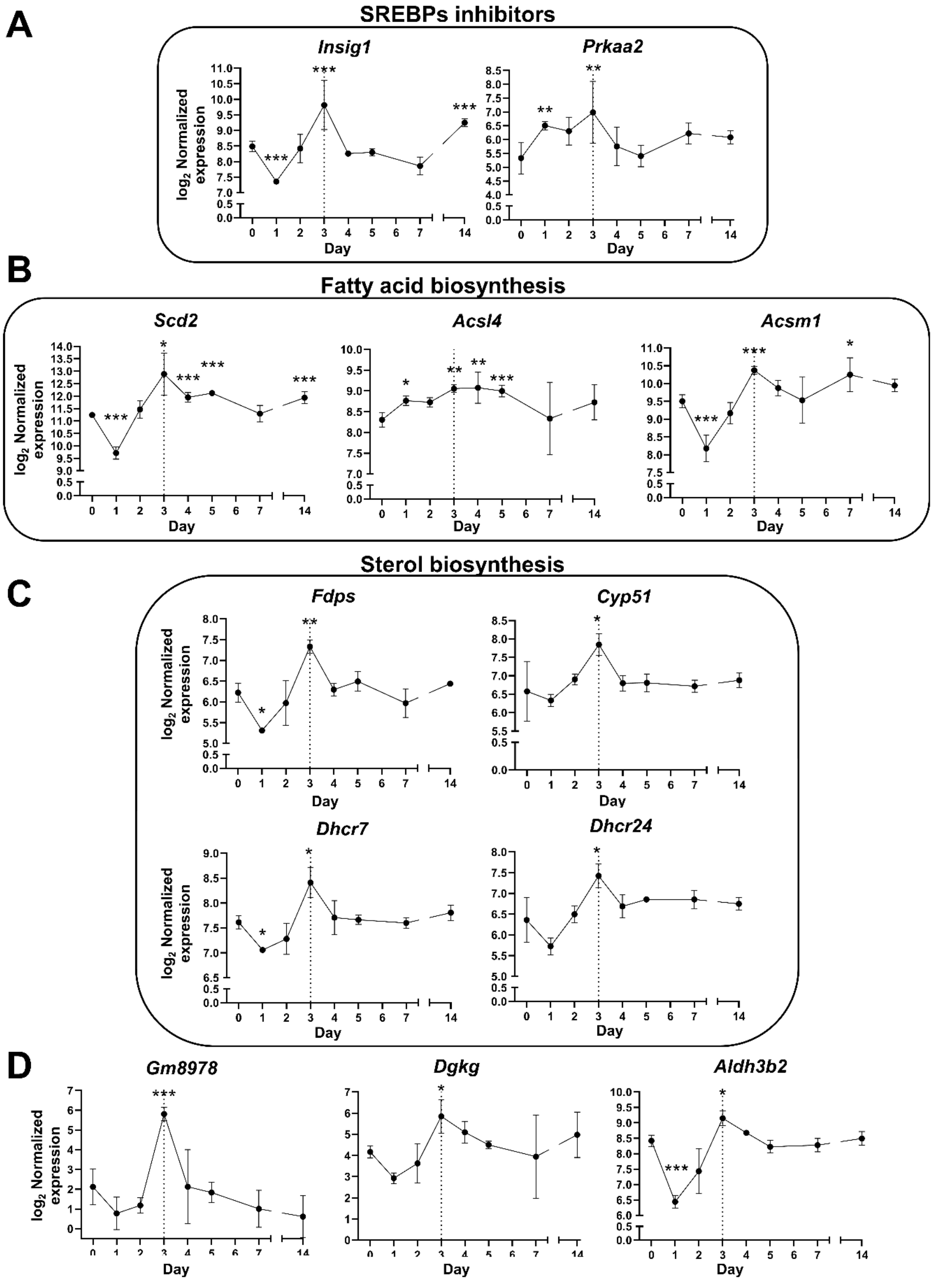

Fig. S7: Normalized mRNA expression of genes involved in (A) the regulation of SREBP-1, (B) fatty acid biosynthesis, (C) sterol biosynthesis, and (D) the modification of lipids in LGs injured by IL-1α injection. Error bars show the mean (SD). Statistical significance of $\log_2$ Fold Change compared to uninjured control (day 0) is shown with: * P-value adj. < 0.05; ** P-value adj. < 0.01; *** P-value adj. < 0.001.

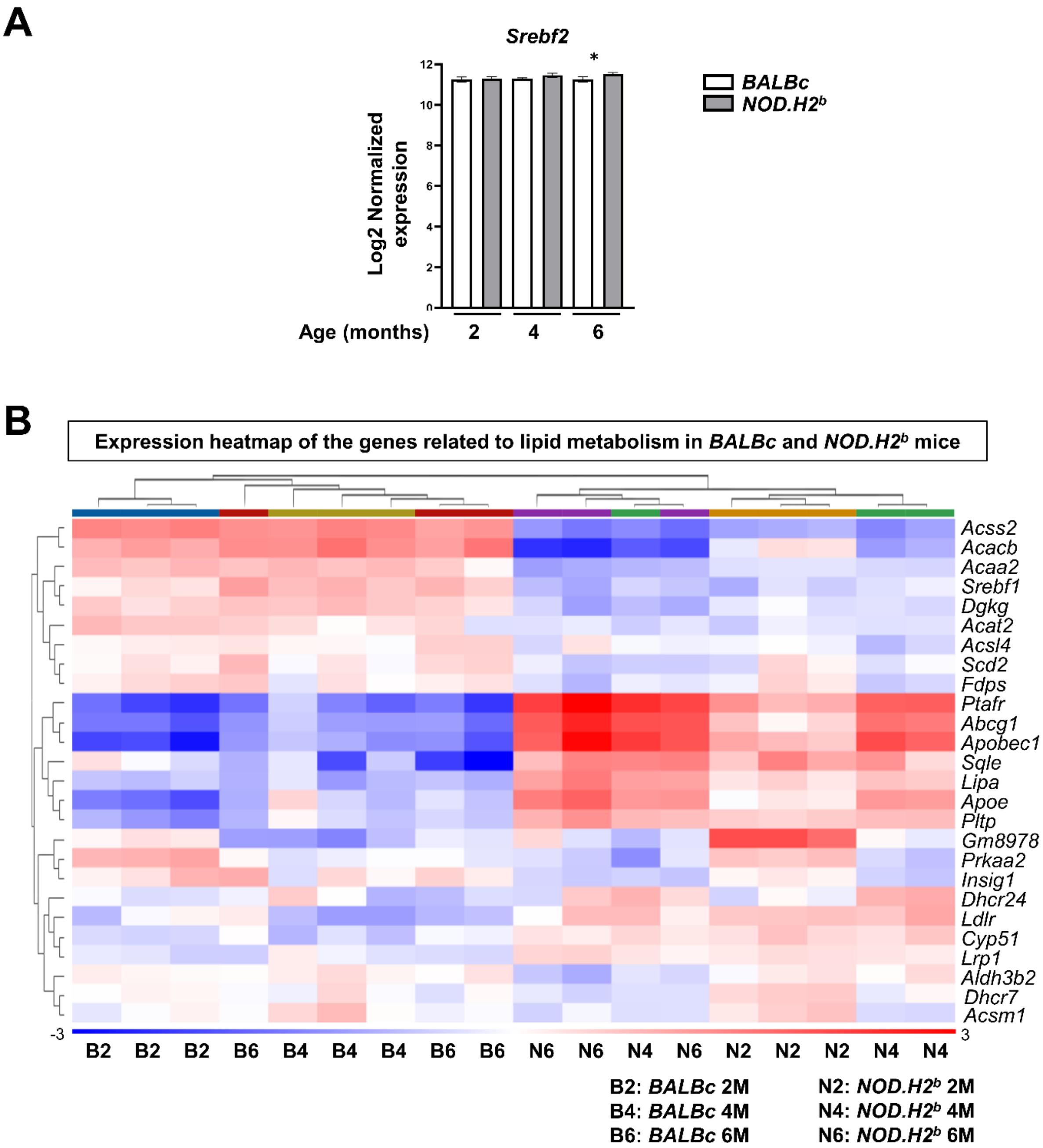

Fig. S8: (A) Normalized expression of *Srebf2* in *NOD.H2*^{b} and *BALBc* LGs by RNA-seq. Error bars show the mean (SD). Statistical significance of $\log_2$ Fold Change compared to respective *BALBc* control is shown with: * P-value adj. < 0.05; ** P-value adj. < 0.01; *** P-value adj. < 0.001. (B) Expression heatmap in *NOD.H2*^{b} and *BALBc* LGs of the lipogenic genes significantly upregulated on day 3 after acute LG injury.
